# Target modulation and pharmacokinetics/pharmacodynamics translation of the BTK inhibitor poseltinib for model-informed phase II dose selection

**DOI:** 10.1038/s41598-021-98255-7

**Published:** 2021-09-21

**Authors:** Joo-Yun Byun, Yi T. Koh, Sun Young Jang, Jennifer W. Witcher, Jason R. Chan, Anna Pustilnik, Mark J. Daniels, Young Hoon Kim, Kwee Hyun Suh, Matthew D. Linnik, Young-Mi Lee

**Affiliations:** 1 Hanmi Research Center, Hanmi Pharm. Co. Ltd., 14 Wiryeseong-daero, Songpa-gu, Seoul, 05545 Korea; 2 Lilly Biotechnology Center, 10290 Campus Point Drive, San Diego, 92121 USA

**Keywords:** Drug discovery, Rheumatology

## Abstract

The selective Bruton tyrosine kinase (BTK) inhibitor poseltinib has been shown to inhibit the BCR signal transduction pathway and cytokine production in B cells (Park *et al.* *Arthritis Res. Ther.* **18**, 91, 10.1186/s13075-016-0988-z, 2016). This study describes the translation of nonclinical research studies to a phase I clinical trial in healthy volunteers in which pharmacokinetics (PKs) and pharmacodynamics (PDs) were evaluated for dose determination. The BTK protein kinase inhibitory effects of poseltinib in human peripheral blood mononuclear cells (PBMCs) and in rats with collagen-induced arthritis (CIA) were evaluated. High-dimensional phosphorylation analysis was conducted on human immune cells such as B cells, CD8 + memory cells, CD4 + memory cells, NK cells, neutrophils, and monocytes, to map the impact of poseltinib on BTK/PLC and AKT signaling pathways. PK and PD profiles were evaluated in a first-in-human study in healthy donors, and a PK/PD model was established based on BTK occupancy. Poseltinib bound to the BTK protein and modulated BTK phosphorylation in human PBMCs. High-dimensional phosphorylation analysis of 94 nodes showed that poseltinib had the highest impact on anti-IgM + CD40L stimulated B cells, however, lower impacts on anti-CD3/CD-28 stimulated T cells, IL-2 stimulated CD4 + T cells and NK cells, M-CSF stimulated monocytes, or LPS-induced granulocytes. In anti-IgM + CD40L stimulated B cells, poseltinib inhibited the phosphorylation of BTK, AKT, and PLCγ2. Moreover, poseltinib dose dependently improved arthritis disease severity in CIA rat model. In a clinical phase I trial for healthy volunteers, poseltinib exhibited dose-dependent and persistent BTK occupancy in PBMCs of all poseltinib-administrated patients in the study. More than 80% of BTK occupancy at 40 mg dosing was maintained for up to 48 h after the first dose. A first-in-human healthy volunteer study of poseltinib established target engagement with circulating BTK protein. Desirable PK and PD properties were observed, and a modeling approach was used for rational dose selection for subsequent trials. Poseltinib was confirmed as a potential BTK inhibitor for the treatment of autoimmune diseases.

**Trial registration:** This article includes the results of a clinical intervention on human participants [NCT01765478].

## Introduction

Rheumatoid arthritis (RA) is a chronic autoimmune disease manifested by aggressive synovitis that causes bone and cartilage damage over time^1,2^. The production of abnormal B cells and autoantibodies, primarily anticitrullinated protein antibody (ACPA) and rheumatoid factor (RF), by most RA patients indicate that the function of B cells is involved in RA disease progression. Accordingly, drugs targeting B cells have attracted increasing attention for the treatment of RA.

Bruton tyrosine kinase (BTK) is a TEC family cytoplasmic tyrosine kinase essential for B cell activation mediated through the B cell receptor^3^. Other nonreceptor TEC family kinase (TFK) members include TEC, ITK, BMX/ETK, and TXK/RLK^4^. BTK is expressed by most cells in the hematopoietic system apart from natural killer (NK)^5^ and T cells^6^. However, BTK deficiency most prominently impacts humoral immunity^7^ while largely preserving innate immune functions, as seen in X-linked agammaglobulinemia (XLA) patients carrying BTK mutations^8^. BTK inhibition has a profound impact on B cell function^9^, and recently, its contribution to myeloid cell function downstream of other signaling pathways was demonstrated by the ability of pharmacological inhibitors of BTK to inhibit other TEC family members expressed in myeloid cells to varying degrees^10^. BTK inhibitors have also been shown to exert effects on rodent models of collagen-induced arthritis (CIA) and other inflammatory diseases, providing a rationale for targeting BTK inhibition in autoimmune diseases^1,11–13^.

Herein, we report the structure of poseltinib, a covalent inhibitor that is relatively selective for a conversed cysteine shared by only ten kinases within the kinome. As poseltinib biochemically binds to some of these nine other kinases, we sought to define its inhibition fingerprint in various immune cells, such as B cells, T cells, monocytes, NK cells and neutrophils, by high-dimensional phosphoflow cytometry.

Previously, we reported that poseltinib potently inhibits BTK kinase activity and BCR signaling^14^. Here, we also demonstrate that a BTK inhibitor similar to poseltinib is also able to inhibit the antigen-presentation function of B cells to indirectly inhibit T cell activation. Finally, we characterize the pharmacokinetics (PKs) and pharmacodynamics (PDs) of poseltinib in a phase I study in healthy volunteers and use these data to develop computational models for dose selection for phase II studies.

## Materials and methods

### Chemicals

Poseltinib (HM71224/LY3337641) and HM71035/LSN3359180 were supplied by Hanmi Pharmaceutical (South Korea), dissolved in DMSO to produce a 10 mM stock solution for in vitro and ex vivo studies, and stored at 4 °C until use in the indicated assay. For in vivo studies, poseltinib 2HCl was dissolved in DMSO, and then Tween 20 and distilled water were added gradually to achieve the desired concentration (1:0.5:28.5 (*v/v/v*)). Test compounds to be administered to CIA model rats were freshly prepared every day. In the clinical trial, healthy volunteers were orally administered poseltinib tablets in multiples of 5 mg or 20 mg.

### Protein and inhibitor docking modeling

In silico binding analysis of poseltinib and the BTK protein via crystallization was performed. Briefly, BTKHM was purified and concentrated to 8 mg/ml was set up in vapor diffusion sitting drops at a ratio of 1:1 with a well solution of 100 mM Imidazole pH 6.2, 15% Ethylene Glycol, 20% Glycerol, and 10% PEG 4 K. Crystals grew within one day and were soaked overnight in 5 mM solution of Poseltinib. Crystals were flash-frozen in liquid nitrogen without additional cryoprotectant. X-ray diffraction data were collected at the Lilly Research Laboratories Collaborative Access Team (LRL-CAT) beam line at the Advanced Photon Source, Argonne, IL.

### Isolation of human immune cells

Peripheral blood mononuclear cells (PBMCs) were isolated by Ficoll-Hypaque gradient centrifugation using Leucosep tubes (227290, Greiner Bio-one, Austria) with histopaque (10771, Sigma-Aldrich, MO, USA) separation solution. After the mixed solution (whole blood:RPMI1640 media with 10% FBS = 1:1) was poured onto the upper histopaque solution, the cells were harvested by centrifugation at 800 × g for 20 min at room temperature with the brake off. The cells were washed once with DPBS, and subsequently, red blood cells were lysed by resuspending the cell pellet with ACK lysing buffer (A10492-01, Thermo Fisher Scientific, MS, USA).

### Analysis of BTK inhibition in human PBMCs

High-dimensional phosphorylation analysis using Pathway Phenotyping technology (Primity Bio, Fremont, CA) was employed to create a high-resolution map of the effect of poseltinib on signaling pathways in immune cells. The analysis measured 94 intracellular signaling nodes (81 phosphorylation sites, 10 total protein levels and 3 methylation/acetylation sites) in stimulated immune cells, including B cells (IgM plus CD40L), CD4 + and CD8 + T cells (IL-2; CD3 plus CD28), monocytes (M-CSF), NK cells (IL-2), and granulocytes (LPS). PBMCs or whole blood was incubated with poseltinib (30 µM) for 2 h prior to the addition of the stimulant, and the 96 nodes were measured at the single-cell level at 2 min, 5 min, 15 min and 60 min.

### Antigen-presentation assays

DO11.10 and BALB/C mice were purchased from The Jackson Laboratory and housed in accordance with the policies of the IACUC of Eli Lilly and Company. BALB/C mice were primed with 10 µg/ml ovalbumin (OVA) in Alhydrogel (InvivoGen, CA, USA) at a 2:1 ratio and boosted with 10 µg/ml OVA in PBS 2 weeks after priming. BALB/C mice were sacrificed 4 days after boosting, and B cells were purified from splenocytes using CD19 microbeads (Miltenyi Biotec) for positive selection. B cells were preincubated with HM71305/LSN3359180 for 1 h before the addition of OVA protein or OVA323-339 peptide, and T cells were positively isolated from DO11.10 transgenic mice using CD4 microbeads (Miltenyi Biotec). The supernatants were collected after 18 h, and IL-2 was measured by ELISA using mouse IL-2 DuoSet (DY402, R&D Systems). FACS analysis of CD69 (H1.2F3, Biolegend), CD86 (B7-2, BD Biosciences) and H2-IA/IE (M5/114.15.3, BD Biosciences) was performed using BD LSR Fortessa, and data were analyzed by FlowJo10 (FlowJo, LLC).

### Biochemical assays

The kinase activity of BTK was measured using the Z′-LYTE assay based on fluorescence resonance energy transfer (FRET). Z′-LYTE Tyrosine 01 (PV3190, Thermo Fisher Scientific) and recombinant human BTK (PV3363, Thermo Fisher Scientific) were processed in black 384-well plates (#3575, Corning, NY, USA) according to the manufacturer’s instructions. Briefly, BTK, peptide, ATP and inhibitor mixtures were incubated for 1 h at room temperature. Then, Development Reagent A was added to each well, and incubation proceeded for another 1 h. The reaction was stopped by the addition of stop solution, and the final fluorescence was detected at 445 nm (Coumarin Emission) and 520 nm (Fluorescein Emission) by a GeminiEM Microplate Reader (Molecular Device, SpectraMAX, CA, USA). The data were analyzed by GraphPad Prism 6 (GraphPad Software, CA, USA) to determine the IC_50_ values.

### Occupancy assay

A total of 5 × 10^5^ Ramos cells/well were plated in 96-well PDL-coated plates in DMEM with high glucose (GIBCO) supplemented with 10% FBS. Serial dilutions of LY3439943 in DMSO (final DMSO concentration, 0.4%) were incubated with the cells for 1 h at 37 °C. An equal volume of a covalent biotinylated BTK probe was added (final concentration, 167 nM), and the cells were incubated for another 1 h at 37 °C. The cells were lysed, and the amount of BTK was determined using the AlphaScreen IgG Detection Kit (Perkin Elmer 6760617R) and an anti-BTK antibody (Thermo Scientific Cat# MA5-14929) following the manufacturer’s instructions. The signal was measured at 615 nm with an Envision plate reader (Perkin Elmer). The absolute IC_50_ was calculated as percent inhibition of the signal window as defined by the maximum (assay medium with 0.4% DMSO) and minimum (1 µM ibrutinib, a known BTK inhibitor) using a 4-parameter nonlinear logistic equation (GeneData Screener 13.0.5 or GraphPad Prism 7.03).

### Cloning and crystallization of BTK

Residues 391–659 of BTK were PCR amplified, and TOPO was cloned into the pFastBac (KF) vector (Life Technologies). Expression in this vector generated BTK residues 391–659 fused to a TEV cleavable N terminal 6X histidine tag. Fermentation of BTK in *Sf*9 cells was performed for 48 h, then the cells were harvested by centrifugation, and the pellets were stored at − 80 °C for purification.

Protein was concentrated to 8 mg/ml in vapor diffusion sitting drops at a ratio of 1:1 with a well solution of 100 mM imidazole, pH 6.2, 15% ethylene glycol, 20% glycerol, and 10% PEG 4 K. Crystals grew within one day and were soaked overnight in a 5 mM solution of poseltinib. The crystals were flash-frozen in liquid nitrogen without additional cryoprotectant. X-ray diffraction data were collected at the Advanced Photon Source (Argonne, IL).

### Collagen-induced arthritis model or rat

All animals were obtained from Charles River Japan (150 ± 10 g, 6 weeks old). The experimental protocols were approved by the animal care and use committee of the Hanmi Research Center and performed in accordance with approved guidelines. Briefly, 7-week-old male Lewis rats were immunized with a collagen emulsion of equal volumes of IFA (F5506, Sigma-Aldrich) and bovine type II collagen (total volume, 0.6 mL) via intradermal injection into the base of the tail. Seven days later, the rats were given a booster immunization of 0.3 ml of collagen emulsion in the same manner. The incidence of arthritis was 79% on day 6 after booster immunization, and the rats were randomized into 4 groups when the average clinical score of each animal was 2.8 (on a scale of 16) (n = 20 in each drug-administered group, n = 8 in the CIA control group). Poseltinib was orally administered once a day for 9 consecutive days at a dose of 0.3, 1, or 3 mg/kg. The arthritis score was determined by grading each paw from 0 to 4 based on erythema, swelling, and flexion of the joint. Body weight was also measured three times per week.

### Histopathological assessment of arthritis

Briefly, the hind legs of each rat were fixed with 10% formalin, decalcified in 5% formic acid, embedded in paraffin, and cut for pathological staining. Arthritis, bone erosion and synovitis were determined by hematoxylin and eosin (H&E) staining, and cartilage damage was evaluated by safranin O staining. All parameters were scored on a scale of 0 to 4 based on severity according to the following criteria: 0 = no erosion, 1 = mild (focal subchondral erosion), 2 = moderate (multiple subchondral erosions), 3 = high (multiple subchondral erosions and focal erosion of the talus), and 4 = maximum (multiple erosions of the tarsal and metatarsal bones). This test was conducted by the Department of Pathology of Asan Medical Center (University of Ulsan College of Medicine, Republic of Korea).

### Clinical trial study design

The first-in-human study of poseltinib was designed as a randomized, placebo-controlled and double-blind [NCT01765478] clinical trial aimed at determining the safety, tolerability, PKs, PDs, and food effect of poseltinib in healthy adult male volunteers. This study consisted of 3 parts: (1) a single ascending dose (SAD) study in which two groups of 9 subjects each (N = 18) were administered escalating single oral doses of poseltinib (10 mg, 20 mg, 40 mg, 80 mg, 140 mg, and 200 mg) or placebo under fasting conditions in a randomized, double-blind, alternating panel fashion; (2) a single food effect study in which eight subjects (N = 8) were administered an open-label, single oral dose of poseltinib (60 mg) in a 2-sequence, crossover design; and (3) a multiple ascending dose (MAD) study involving eight groups of 8 subjects each (N = 64), with 48 subjects receiving multiple oral doses of poseltinib (n = 6 per group) and 16 receiving placebo (n = 2 per group) under fasting conditions for 14 consecutive days in a randomized, double-blind fashion. Five groups received poseltinib (10 mg, 20 mg, 40 mg, 80 mg, or 120 mg) once daily (QD), while 3 groups received poseltinib (5 mg or 20 mg, 40 mg or 60 mg) twice daily (BID).

PK/PD data from 24 subjects who had received the drug BID among the 90 total subjects who participated in the MAD study were analyzed. Blood was collected on day 1 before drug administration and 1 h, 2 h, 4 h, and 12 h after drug administration. Blood samples were taken 3 days and 2 h after drug administration. Additional blood samples were taken on day 6 (5, 20, and 60 mg/kg), day 15 (40 mg/kg), day 17, and day 21 for PK/PD analysis.

### Quantitative analysis of pharmacodynamics

The BTK occupancy assay and analysis of BTK phosphorylation inhibition were performed as described previously^1^. Briefly, isolated PBMCs were lysed with RIPA buffer (R0278, Sigma-Aldrich) containing 1 × protease/phosphatase inhibitor (1861281, Thermo Fisher Scientific) and EDTA (1861274, Thermo Fisher Scientific). The supernatants were collected by 10 min at 14,000 × *g* in microcentrifuge at 4 °C and biotinylated probe was added to bind free BTK for 1 h. Rabbit anti-BTK Ab (1:5000 in 1% BSA) bound the captured BTK/probe complex, and was detected with anti-Mouse antibody conjugated with HRP then ultra-TMB solution was added to each well for the free BTK analysis. Analysis of free and total BTK levels for the phase I study was conducted by Cambridge Biomedical Inc. (MA, USA). BTK occupancy was calculated by taking the free BTK and total BTK results from a given donor and used the following formula:

$$ \% {\text{BTK}}\;{\text{occupancy}} = 100\% - \left( {{\text{Free}}\;{\text{BTK/total}}\;{\text{BTK}}} \right) \times 100. $$

For analysis of BTK phosphorylation inhibition, the relative percentage of phosphorylated BTK was calculated by the following equation: phospho-BTK level / total BTK level × 100. The results are presented as the mean percentage ± S.D.

### Analysis of PK parameters of preclinical and human subjects

After drug administration, whole blood was collected from CIA model rats and human subjects at the indicated times. Plasma was obtained by centrifugation at 12,000 rpm for 2 min and stored at − 20 °C until analysis. The poseltinib concentration in the plasma was analyzed by UPLC H-class (Waters, MS, USA) with Xevo TQ tandem mass spectrometry (Waters), and PK parameters were calculated by noncompartmental analysis using Phoenix WinNonlin 6.3 (Pharsight, CA, USA).

The PKs of poseltinib in humans were further analyzed by nonlinear mixed effects modeling (NONMEM; version 7.4.2, ICON Development Solutions, Ellicott City, Maryland). A PK model was developed using single- and multiple-dose PK data from the human study. The model included two compartments, a transit compartment absorption model, intersubject variability of key parameters, and residual error.

### PK/PD model development for human subjects

The free BTK data from the BID-treated cohorts of the MAD portion of the human study were incorporated into the model and used to describe the covalent binding of poseltinib to free BTK. A free BTK compartment, which had a zero-order formation rate and a first-order clearance rate, was included in the PK/PD model. An irreversible binding parameter was included to describe the binding rate of poseltinib and free BTK. The bound BTK, which was produced as a result of binding with poseltinib, was assumed to be cleared at the same rate as free BTK. The percentage BTK occupancy was calculated post hoc based on the assumption that free BTK at baseline represented total BTK.

### Application of a quantitative systems pharmacology (QSP) model for phase II dose selection

The PK/PD model for poseltinib and BTK occupancy described above was incorporated into a QSP model for RA (PMID: 16986268). In vitro BTK occupancy assays were used as bridges to estimate the equivalent in vitro concentrations of poseltinib in the PD assays that corresponded to the occupancy obtained at steady state for the clinical doses. E_max_ models were constructed to estimate inhibition levels for poseltinib across three canonical BTK-related pathways in the models of B cell activation (from CD69 expression in B cells), FcR activation (from plate-bound IgG-stimulated cytokine production in THP-1 cells), and TLR activation (from LPS-stimulated THP-1 cells) in addition to off-target effects (ITK, from anti-CD3-induced stimulation of IL-17 in PBMCs). IC_50_ values were normalized to determine time dependence (PMID: 19797607; PMID: 18854379) due to the covalent nature of poseltinib. Virtual populations were selected from a large cohort of virtual patients with variations in parameters relevant to RA such that they fit the phase III clinical trial American College of Rheumatology (ACR) response data from competitor therapies in patients with an inadequate response to methotrexate (PMID: 23841912). Virtual populations were qualified by predicting the outcome for fostamatinib, an SYK inhibitor, which was not used for fitting. Potential dose responses were evaluated by fitting populations to the aforementioned clinical data while maximizing the predicted efficacy of poseltinib under different scenarios to better understand its potential efficacy at lower doses. The scenarios tested included unbiased, maximal efficacy, maximal efficacy with the largest difference between 30 and 10 mg, and maximal efficacy with the smallest difference between 30 and 10 mg. The performance of poseltinib was simulated across a dose range of 1 mg to 40 mg.

### Ethics approval and consent to participate

#### Human experiments

All clinical methods were carried out in accordance with relevant guidelines and regulations. All experimental protocols were approved by the Independent Ethics Committee (IEC) of the “Stichting Beoordeling Ethiek Biomedisch Onderzoek” (“Foundation Evaluation of the Ethics of Biomedical Research”, Stationsstraat 9, 9401 kV Assen, The Netherlands). The study was conducted in accordance with the principles of the Declaration of Helsinki in place at the time of study conduct. It was followed in compliance with the International Conference on Harmonisation (ICH) E6 Guideline for Good Clinical Practice (GCP) (Committee for Proprietary Medicinal Products (CPMP) guideline CPMP/ICH/135/95), and compliant with the European Union Clinical Trial Directive (EU CTD): Directive 2001/20/EC. Informed consent was obtained from all participants.

The information of clinical trial is following:

ClinicalTrials.gov Identifier [NCT01765478; https://clinicaltrials.gov/ct2/show/NCT01765478?term=HM71224&draw=2&rank=1] First screening was 30 Jan. 2013 and no ISRCTN approved.

#### Animal experiments

All experiments were conducted in compliance with the guidelines for the care and use of laboratory animals. In all animal experiments, protocols were approved by the Institutional Animal Care and Use Committee of the Hanmi Research Center, and the procedures were performed according to the approved guideline. All performed animal study was carried out in compliance with the ARRIVE guidelines.

### Consent for publication

We have received consent for publication from all authors.

## Results

### Discovery of poseltinib and the docking module of the BTK protein

**Figure 1:**
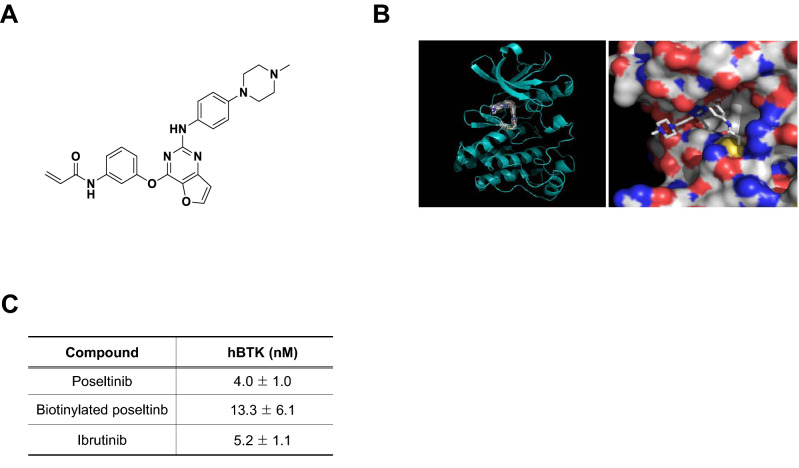
The structure of poseltinib. (**A**) Chemical structure of poseltinib (C_26_H_26_N_6_O_3_ as free base; molecular weight, 470.52). (**B**) In silico analysis of binding between poseltinib and BTK (left) and a docking module within the active site of crystallized BTK (right). (**C**) Biochemical assay of the effects of poseltinib and its biotinylated probe on BTK. The data represent the mean ± S.D (nM).

We identified poseltinib (Fig. 1A) as a potent and selective BTK inhibitor. Poseltinib was rationally designed to possess a high affinity for the ATP binding pocket. In silico binding analysis of poseltinib with BTK revealed a docking module within the active site of crystallized BTK (Fig. 1B). Biochemical analysis BTK binding to poseltinib and the binding of its biotinylated probe to poseltinib revealed IC_50_ values of 4.0 nM and 13.3 nM, respectively. Poseltinib had a comparably robust inhibitory effect on BTK as ibrutinib which approved as BTK inhibitor for anti-cancer therapy (Fig. 1C). Biochemical properties for poseltinib on other kinases, including BMX, TEC and TXK were performed by fluorescence resonance energy transfer (FRET) and showed potent inhibitory effect under 10 nM activity. Poseltinib also showed inhibitory effect on ITK or JAK3 but selectivity toward BTK were 53 × and 7.5×, respectively (Supplementary Table 1).

### Poseltinib inhibited BTK kinase activity and regulates BTK-dependent immune cell crosstalk

**Figure 2:**
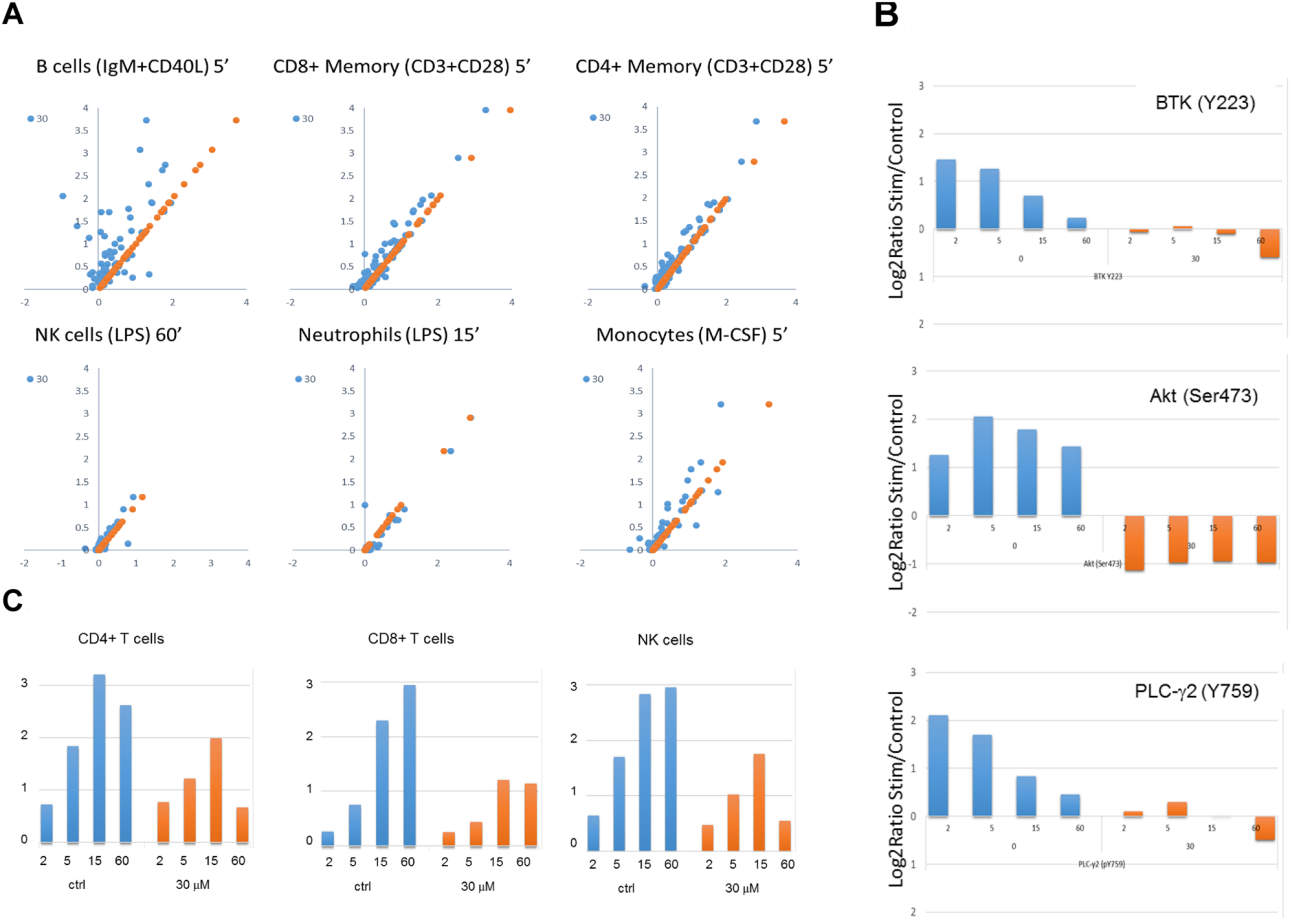
High-dimensional analysis of BTK-dependent phosphorylation in stimulated immune cells revealed a preferential impact on B cells. (**A**) Scattergrams of 96 nodes in the absence (orange) or presence (blue) of 30 µM poseltinib in B cells, CD8 + memory T cells, CD4 + memory T cells, NK cells, neutrophils and monocytes 15 min following stimulation. B cells, T cells and neutrophils showed multiple nodes with substantial stimulation, with poseltinib having the largest impact on B cells. On scattergram for PO4 phospho fingerprint analysis, X and Y axis indicates “Log2 ratio stimultated/control” (**B**) Poseltinib-mediated inhibition of phosphorylation of BTK (Y223), AKT (S473), and PLCγ2 (Y759) in B cells after 2, 5, 15, 60 min treatment. (**C**) Poseltinib-mediated inhibition of IL-2 induced STAT-5 phosphorylation in CD4 + T cells, CD8 + T cells and NK cells after 2, 5, 15, 60 min treatment.

Based on our findings suggesting that poseltinib inhibits BTK in B cells, we examined the impact of poseltinib on human immune cells using high-dimensional analysis of 94 nodes in stimulated immune cells^1^ (Supplementary Table 2 and 3). Scattergrams of cells stimulated in presence or absence of 30 μM poseltinib showed that intervention had profound effects on multiple nodes in B cells stimulated with anti-IgM plus CD40L and less impact on anti- CD3 with CD28 stimulated CD8 + T cells or CD4 + T cells, LPS-induced NK or neutrophils activation, and M-CSF stimulated monocytes (Fig. 2A). In B cells, 30 μM poseltinib inhibited the phosphorylation of BTK (Y223), AKT (S473) and PLCγ2 (Y759) at all time points (Fig. 2B). The inhibitory effect of poseltinib on AKT phosphorylation (S473) was specific to stimulated B cells, as it did not inhibit AKT phosphorylation in monocytes stimulated with M-CSF (data not shown). The effect of poseltinib on JAK3 signaling was observed in stimulated CD4 + T cells, CD8 + T cells and NK cells, in which poseltinib (30 μM) inhibited IL-2-stimulated phosphorylation of STAT5 (Y694) (Fig. 2C).

### HM71035, a close analog of poseltinib, inhibited the antigen-presenting cell function of B cells through inhibition of costimulation

**Figure 3:**
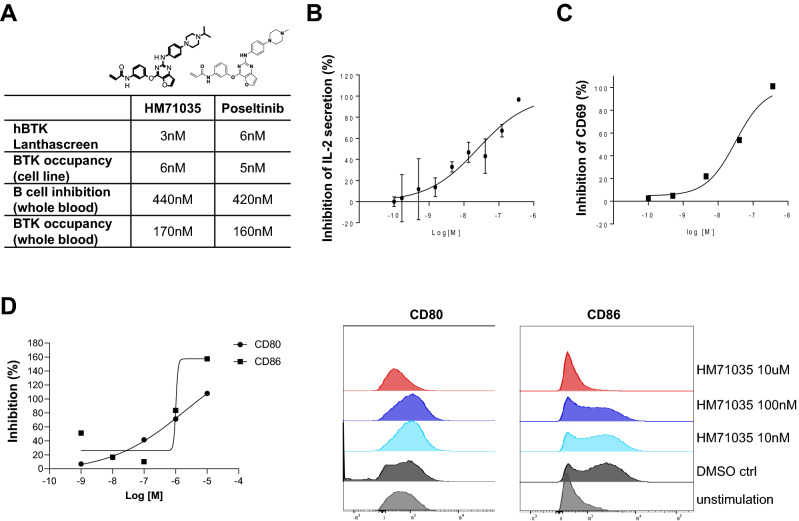
Effects of HM71035 on B cell antigen-presentation function. (**A**) Biochemical activity on hBTK, B cell inhibitory efficacy, and BTK occupancy in B cell line or whole blood of HM71035 and poseltinib. (**B**) B cells that were preactivated in vivo were isolated and incubated with HM71035 for 1 h before being used as antigen-presenting cells to OVA-323–339-specific transgenic T cells. Activation of T cells was inferred from IL-2 production and (**C**) CD69 upregulation, which were inhibited by HM71035. (**D**) Expression levels of the costimulatory markers CD80 and CD86 on B cells were inhibited by HM71035 in a dose-dependent manner.

HM71035/LSN3359180 has a similar chemical structure as poseltinib and had comparable inhibitory effects on BTK in vitro, exerted similar effects on the cellular activity of B cells and showed comparable BTK occupancy in whole blood ex vivo as poseltinib (Fig. 3A). To determine the impact of BTK inhibition on B cell antigen-presenting function, B cells were preactivated in vivo through immunization with OVA antigen then reactivated was done in vivo by boosting with OCA protein 2 weeks after initial OVA protein Alhydrogel immunization. And then reactivated B cells were cocultured with naïve DO11.10 transgenic T cells in the presence of various concentrations of HM71035/LSN3359180 and OVA protein or and OVA323-339 peptide. B cell-dependent T cell activation was reduced in a dose-dependent fashion by HM71035, as reflected by the reductions in IL-2 secretion (Fig. 3B) and CD69 expression on T cells (Fig. 3C). In this experimental setting, the B cells are exposed to the peptide antigen in vitro, so there is further activation through the BCR. The preactivation of B cells in vivo induced high levels of MHC II expression, but HM71035 was still able to suppress the expression of CD80 and CD86 (Fig. 3D). 100 nM was roughly the IC_50_ effect on the B cells, which correlated to that of IL-2 and CD69 inhibition.

### Treatment with poseltinib resulted in dose-dependent reductions in arthritis severity and the histological index in CIA model rats

**Figure 4:**
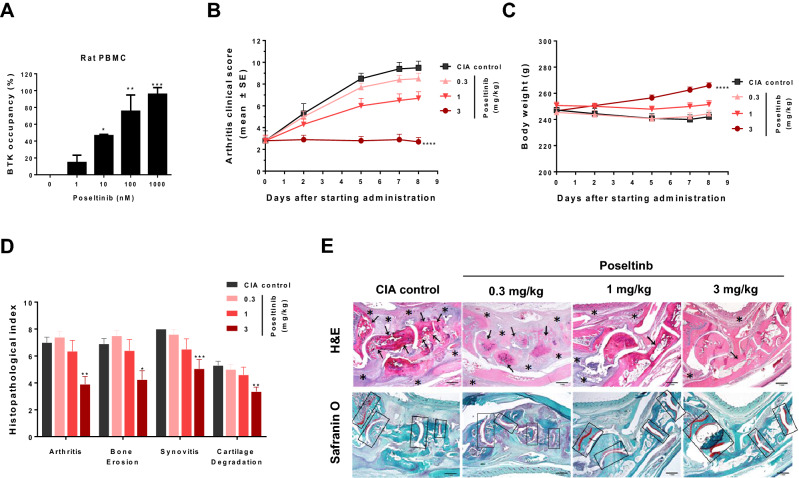
Dose-dependent inhibition of arthritis severity by poseltinib in a rat model of collagen-induced arthritis (CIA). (**A**) Normal rat PBMC were treated with poseltinib at the indicated concentrations for 1 h, and then free BTK was detected using a biotinylated poseltinib probe, as described in the Materials and Methods. BTK occupancy (%) was calculated from the free BTK percentage, which was compared with that of the untreated control group. The bars represent the ± S.D of independent experiments. *p < 0.5, ** *p* < 0.1 and *** *p* < 0.001 vs. the nontreated control group. (**B**–**E**) CIA-induced rats were administered the indicated dose of poseltinib for 9 days starting 6 days after injection of type II collagen emulsion. (**B**) The clinical scores and (**C**) body weight loss of rats treated with the drug for 9 days were estimated. (**D**) Histopathological index analysis of both hind legs was conducted on the final day to assess ankle joint damage. (**E**) Representative images of H&E and Safranin-O staining of the hind legs in CIA model rats taken under a light microscope for histopathological assessment. Bone erosion (** →**) was quantified a subchondral and bone destruction and synovitis (*) was scored degree of pannus formation, including synovial inflammation, hypertrophy, interstitial edema, and fibrosis (H&E). Cartilage damage (□) was semiquantitatively scored as an intensity of Safranin-O staining (red). The data are presented as the mean ± SEM. *p < 0.5, ** *p* < 0.1 and *** *p* < 0.001, **** *p* < 0.0001 vs. the CIA control group (one-way ANOVA for **C**; Kruskal–Wallis test for **B**,**D**).

The in vitro binding of poseltinib to the BTK protein was assessed in rat PBMCs. We performed a molecular probe assay of poseltinib, which showed potent BTK target modulation with an IC_50_ of 14.6 ± 3.3 nM (Fig. 4A). In our previous study, we examined the in vivo efficacy of poseltinib in a mouse CIA model (Arthritis Research & Therapy volume 18, Article number: 91, 2016). Here, we explored whether poseltinib is also efficacious in a rat CIA model. Oral administration of poseltinib significantly reduced arthritis scores in a dose-dependent manner. Notably, 3 mg/kg poseltinib had a significant antiarthritic effect (p < 0.0001 versus CIA controls, Kruskal–Wallis test). At 3 mg/kg, poseltinib prevented weight loss induced by the disease (Fig. 4C). However, treatment effect with 0.3 mg/kg poseltinib had the minimum on reducing arthritis scores (Fig. 4B). Poseltinib dose-dependently reduced the degree of histopathologic abnormality in the ankle joints of CIA rats. Moreover, 3 mg/kg poseltinib improved the arthritis index (44% reduction, ^**^ *p* < 0.01), bone erosion index (38% reduction, ^*^ *p* < 0.05), synovitis index (37% reduction, ^***^ *p* < 0.001) and cartilage degradation index (37% reduction, ^**^ *p* < 0.01) (Fig. 4D). Representative staining of cartilage tissue is shown in Fig. 4E. Treatment with 1 mg/kg and 3 mg/kg poseltinib, but not 0.3 mg/kg poseltinib significantly inhibited bone cartilage disruption. Poseltinib significantly suppressed CIA in rats in a dose-dependent manner, indicating a correlation between target occupancy and disease modification. These data provide that a novel BTK inhibitor poseltinib showed a good feasibility as BTK inhibitor for preclinical CIA rat model.

### Relationship between PKs/PDs and efficacy in CIA model of rat

**Figure 5:**
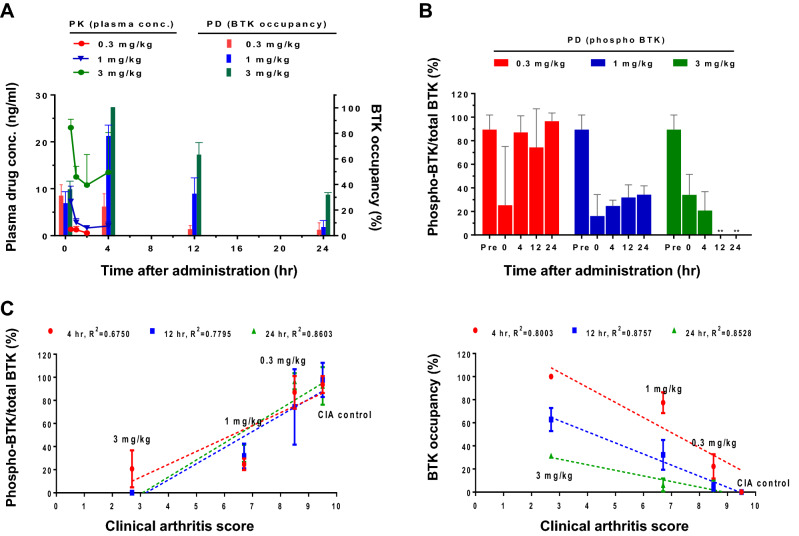
Pharmacologic inhibition of BTK and its correlation with efficacy. (**A**,**B**) Whole blood was collected from the CIA-induced rats at the 0, 4, 12, 24 h after final drug administration, and then PBMCs and plasma were isolated. BTK phosphorylation was detected by immunoblotting and normalized to the total BTK level, and BTK occupancy was measured by ELISA using a biotinylated probe as described in the Materials and Methods. The concentration of poseltinib in the plasma was below the LLOQ at 4 h after administration. Full-length blots are presented in Supplementary Fig. 1. (**C**) Correlations between drug efficacy (clinical arthritis score) and the PD markers BTK phosphorylation percentage (left)/drug efficacy and BTK occupancy percentage (right). Linear regression was performed by using GraphPad Prism 6. The data are presented as the mean ± S.D.

We next explored the relationship between PKs/PDs and efficacy in a rat model of CIA. Treatment with poseltinib resulted in dose- and time-dependent BTK occupancy and inhibition of BTK phosphorylation in splenocytes from CIA model rats. In addition, 3 mg/kg poseltinib completely occupied on BTK at 12 and 24 h (Fig. 5A) whereas 0.3 and 1 mg/kg poseltinib partially inhibited BTK phosphorylation at the same time points (Fig. 5B). Notably, 3 mg/kg poseltinib demonstrated full BTK occupancy for 4 h after the last administration, whereas 0.3 and 1 mg/kg of poseltinib resulted in partial BTK occupancy at that time point. For PD analysis, phosphorylation of BTK was inhibited by poseltinib in a dose-dependent manner over 24 h.

For PK analysis, plasma samples were obtained on day 9 after the last administration, and the levels of poseltinib were measured. The plasma concentration at 4 h after drug administration was below the lower limit of quantification (LLOQ) (< 0.5 ng/ml) in all treatment groups. After oral administration of 0.3, 1, and 3 mg/kg poseltinib, the AUC_last_ (ng·hr/ml) was 2.0 ± 1.8, 10.0 ± 3.6 and 49.9 ± 10.4 respectively, the C_max_ (ng/ml) was 1.5 ± 0.5, 7.3 ± 3.3 and 22.6 ± 2.3, respectively, and the T_max_ (hr) was 0.6 ± 0.3, 0.5 ± 0.0 and 1.4 ± 1.8, respectively. The AUC_last_ and C_max_ of poseltinib increased nonproportionally (1.0:5.0:25.0 for AUC_last_, 1.0:4.9:15.1 for C_max_) with an increase in dose (1.0:3.3:10.0) from 0.3 to 3.0 mg/kg. The estimated proportionality coefficients (β1) for AUC_last_ and C_max_ were 1.48 (90% confidence interval (CI): 1.25–1.71) and 1.23 (90% CI 1.07–1.40), respectively, and the acceptance range of 90% CI was 0.90–1.10 based on a power model (Table 1). Therefore, poseltinib exhibited nonproportional PKs over the dose range of 0.3–3 mg/kg (Fig. 5B). The correlation between clinical arthritis score and BTK phosphorylation as a PD marker was analyzed, and moderately positive correlations with correlation coefficients of 0.6750, 0.7795 and 0.8603 were observed at 4, 12 and 24 h, respectively (Fig. 5C, left). Arthritis scores were compared with BTK occupancy, and moderately negative correlations with correlation coefficients of 0.8003, 0.8757 and 0.8528 were found at 4, 12 and 24 h, respectively (Fig. 5C, right). Given that the PD biomarker p-BTK correlated with improvement in arthritis severity scores in rats, it may be used to estimate clinical improvements in patients treated with poseltinib.

**Table 1:** PK/PD and efficacy relationship in CIA model of rat.

Parameter		CIA control	Poseltinib		
			0.3 mg/kg	1 mg/kg	3 mg/kg
PD	Arthritis clinical score	9.5 ± 0.6	8.5 ± 0.5	6.7 ± 0.6	2.7 ± 0.4
	Histological assessment (Means of the 4 indexes)	6.8 ± 0.2	6.9 ± 0.2	6.3 ± 0.2	4.1 ± 0.2
PK	AUC_last_ (ng·hr/ml)	-	2.0 ± 1.8	10.0 ± 3.6	49.9 ± 10.4
	C_max_ (ng/ml)	-	1.5 ± 0.5	7.3 ± 3.3	23.6 ± 2.3
	T_max_ (hr)	-	0.6 ± 0.3	0.5 ± 0.0	1.4 ± 1.8

In vivo PK/PD profiles on day 8 (PD) and 9 (PK) after consecutive daily oral administration of poseltinib to CIA model of rats.

Data are presented as the mean ± SEM (PD) and mean ± SD (PK).

Summary of PK/PD profiles of poseltinib in rats CIA model after consecutive daily oral administration. Blood was collected from the treated CIA model rats for PK analysis on day 9 and for PD analysis on day 8.

### Human clinical PK/PD relationship with poseltinib

**Figure 6:**
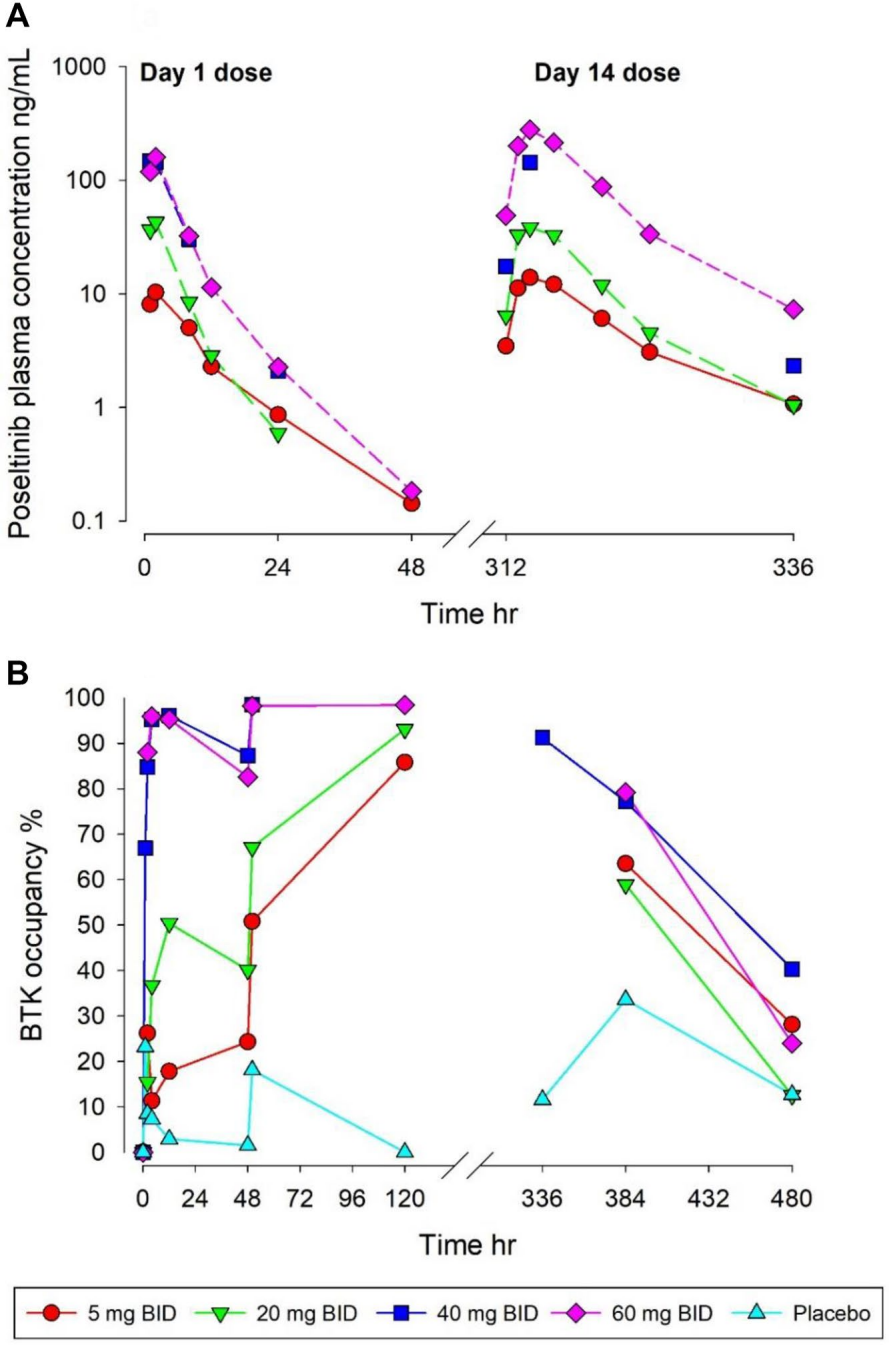
PK/PD relationship of poseltinib in healthy volunteers. (**A**) Healthy volunteers were administered for 5, 20, 40, or 60 mg/kg poseltinib twice a day (BID). Whole blood was collected 0, 1, 2, 8, 12, 24, and 48 h after drug administration for PK analysis. The data are shown as the mean ± S.D. (**B**) BTK occupancy (%) by measuring free BTK from human PBMC lysate was measured by ELISA at the indicated times. Each dot indicates an individual subject, and the lines on the dot plots indicate the mean values. Data from 18 of the 24 subjects from the healthy donor study, excluding 6 participants who were administered placebo, are shown.

**Figure 7:**
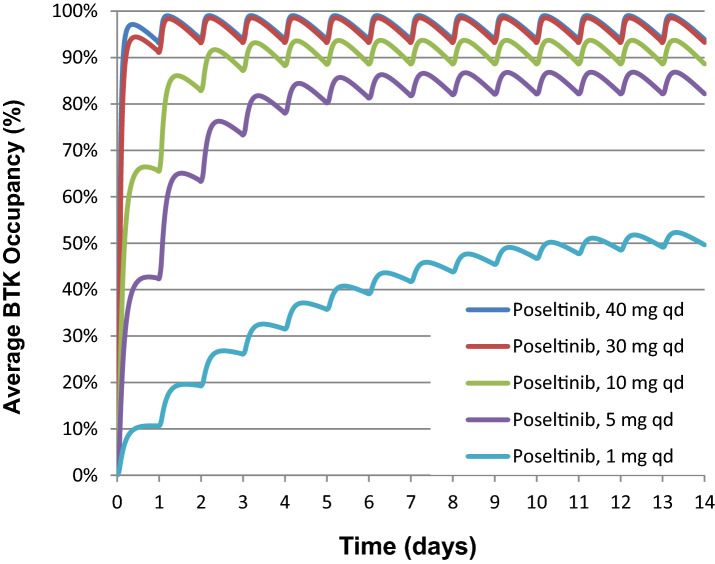
Simulations based on the PK/PD model constructed from phase I trial data from human volunteers illustrating predicted occupancy as a function of time for 1 to 40 mg QD doses of poseltinib.

A PK/PD model of target occupancy was constructed based on the data in Fig. 6. Geometric mean Cmax and AUC0-tau showed an increase with dose and were higher after multiple dosing for 13 days (Fig. 6A, right) than after a single dose (Fig. 6A, left) indicating accumulation of HM71224 after multiple doses. Increase of Cmax, AUC0-last and AUC0-inf was found to be dose proportional after multiple bid doses of HM71224. HM71224 was absorbed with a similar Tmax after all dose levels ranging from 1.00 to 2.00 h after multiple doses. For target engagement on BTK by poseltinib, free BTK level in human PBMCs were measure by ELISA. BTK inhibition by HM71224 is quick with an inhibition of > 90% achieved at 4 h after the first dose for doses of 40 mg HM71224 and higher. A maximum inhibition was observed at 4 h for 60 mg HM71224 and at 12 h for 40 mg HM71224 (Fig. 6B). BTK inhibition > 90% was achieved at steady state (120 h) for doses of 20 mg HM71224 bid and higher. Simulations were used to illustrate the relationship between dose and target occupancy upon daily administration (Fig. 7). The relationship between occupancy and inhibition of BTK-dependent pathways is summarized in Supplementary Table 1. Briefly, inhibition of BCR was measured by inhibition of CD69 upregulation following dextran-anti-IgD stimulation. Immune complex stimulation of TNFa production by monocytes was used to understand FcR-mediated signaling. Anti-CD3 and anti-CD28 stimulation of human PBMCs was used to look at effect of Poseltinib on T cells.

**Figure 8:**
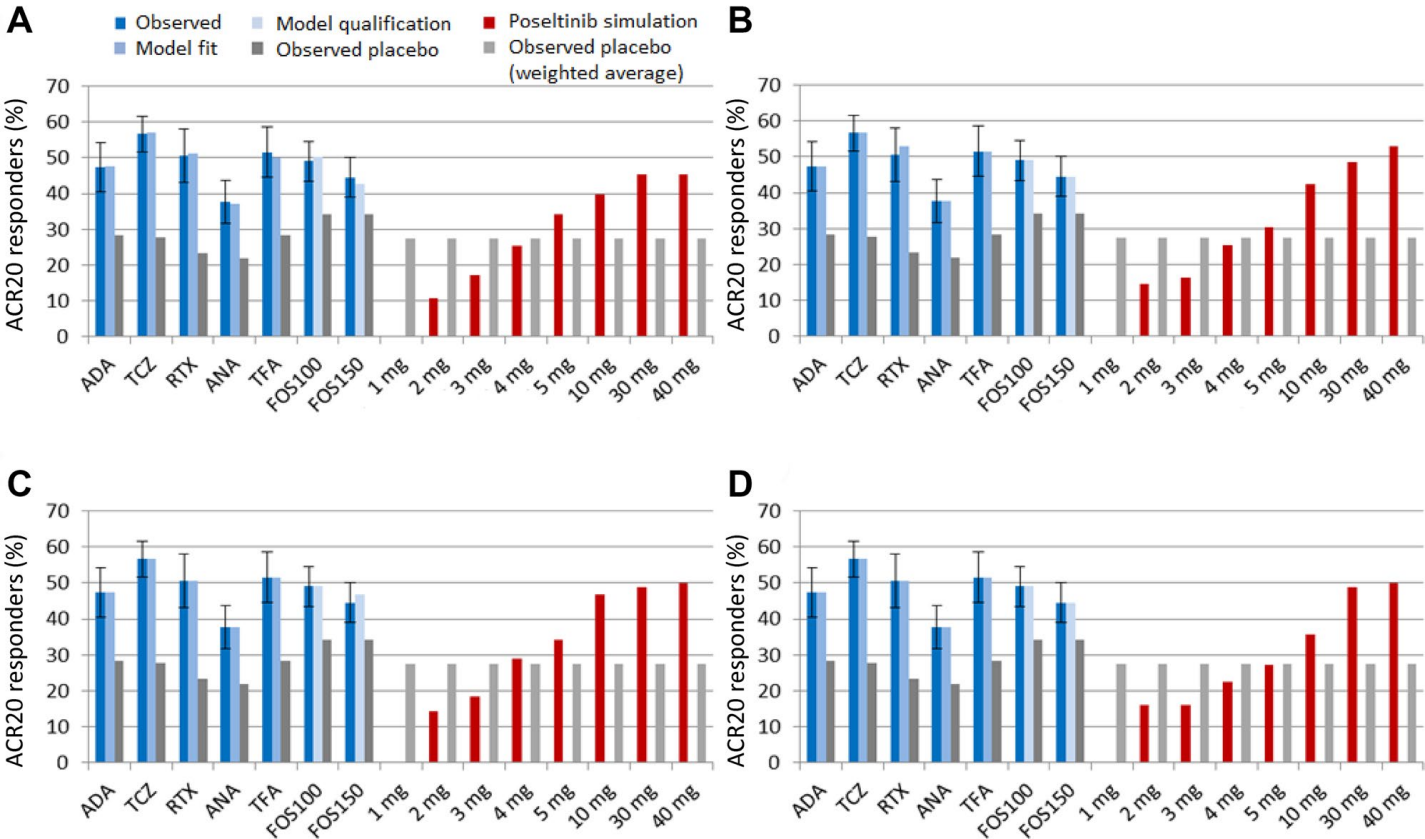
Simulated clinical efficacy (ACR20 responders, %) of poseltinib. Virtual populations in a QSP model for RA were fit to observed clinical trial data (dark blue) for therapeutics with different mechanisms of action. The model was qualified by predicting the clinical response to fostamatinib (FOS, light blue), which was not used for model calibration. The error bars represent the 95% CI of the observed data (see the Supplemental Table 1 for trial details). Virtual populations were considered acceptable if they were within the 95% CI of the observed data. With respect to poseltinib response (red), virtual populations were either unbiased (**A**), biased to exhibit a maximal response to 30 mg (**B**), biased to exhibit a maximal response to 30 mg with minimal difference from 10 mg (**C**), or biased to exhibit a maximal response to 30 mg with maximal difference from 10 mg (**D**). Various doses of poseltinib were simulated and compared against the weighted average placebo response (light gray) in the clinical trials used for model calibration and qualification (Supplemental table 2). *ADA* adalimumab, *TCZ* tocilizumab, *RTX* rituximab, *ANA* anakinra, *TFA* tofacitinib, *FOS100* 100 mg fostamatinib, *FOS150* 150 mg fostamatinib.

ACR scores were simulated in the QSP model for dosing regimens of interest for subsequent studies. Across all virtual population scenarios where poseltinib was predicted to be effective, the effect of 1 mg poseltinib was not predicted to be different from that of the placebo (Fig. 8). By contrast, simulations suggested that 5 mg could be the minimally effective dose. This information was used to select doses for the phase II study in RA patients (NCT02628028).

## Discussion

BTK is highly expressed in B cells and has a critical function in BCR-mediated cell activation and survival^15^. BTK in B cells is of considerable interest as a drug target in autoimmune diseases such as rheumatoid arthritis due to its role in regulating immune tolerance^16^. Previously, we demonstrated that the irreversible BTK inhibitor poseltinib dose-dependently inhibits B cell activity and osteoclast formation in vitro and in vivo^1^ and has an effect in a mouse model for lupus^14^. Poseltinib has also been shown to reduce cytokine production and disease severity in a mouse model of CIA. In this paper, we first report the structure and target modulation properties of poseltinib and also demonstrate correlations between PK/PD parameters and efficacy in preclinical and clinical settings.

B cells are important antigen-presenting cells, especially in the context of limiting antigen concentrations as the immune response progresses, particularly in tissue microenvironments^17^. HM71035, a test compound closely related to poseltinib, inhibits the costimulatory functions of preactivated B cells to impact T cell activation. As opposed to B cell depletion strategies, BTK inhibition functionally inactivates the antibody-dependent and antibody-independent functions of B cells while largely maintaining B cell numbers^18^. Thus, BTK inhibition could be more advantageous than B cell depleting strategies that provide a niche for self-reactive B cell clones that may have a competitive advantage at reconstitution due to higher BAFF-R levels^19,20^.

Compared to other experimental arthritis models, the CIA model is the most frequently used experimental model to mimic human RA and shares similar disease characteristics, such as synovial immune infiltration, pannus formation, joint destruction, and other clinical, immunological and pathologic features, with RA^21–23^. Moreover, the Lewis rat model of CIA is preferable for PK/PD assessment of RA therapeutics^24^. Therefore, this animal model was selected to evaluate the therapeutic effects of poseltinib in this study. BTK occupancy and the inhibition of BTK phosphorylation in the rat model of CIA were correlated with disease score and bone histopathology in a dose-dependent manner. These two PD markers may help to establish the optimal dose range in human clinical trials and may be used as prognostic PD markers to evaluate target engagement in human RA^13,25–27^. Plasma concentrations of poseltinib were below the LLOQ, but this LLOQ phenomenon can be explained by irreversible poseltinib binding to the BTK active site, which caused more prolonged inhibition of BTK after plasma levels of poseltinib had reached undetectable ranges.

Selecting doses for a phase II clinical trial for a disease such as RA, in which only phase I target engagement data from healthy volunteers are available, remains challenging since there are no relevant clinical outputs (e.g., ACR scores) from which to construct an empirical PK/PD model^27,28^. Dose selection is particularly difficult when constrained by the number of doses that can be practically evaluated in a trial (Supplementary Table 4). By linking PK and phase I BTK occupancy to pathway inhibition in the QSP model, estimates for potential ACR responses can be obtained across a dose range^29^. The QSP model helped to inform the low end of the dose range for the phase II RA study and allowed for a more efficient study design.

## Conclusion

Poseltinib showed a probability as novel BTK inhibitor for treatment of autoimmune diseases. In this study, we first report not only the structure and target modulation properties of poseltinib but also correlations between PK/PD parameters and efficacy in preclinical and clinical settings. Poseltinib was confirmed as a potential BTK inhibitor for the treatment of autoimmune diseases and now we are exploring the possibility for further indication expansion such as multiple sclerosis.

## Supplementary Information

Supplementary Information.

## Abbreviations

BCR: B cell receptor
CIA: Collagen-induced arthritis
BTK: Bruton's tyrosine kinase
PBMCs: Peripheral blood mononuclear cells
PKs: Pharmacokinetics
PD: Pharmacodynamics
PLC: Phospholipase C
ATP: Adenosine triphosphate
SYK: Spleen tyrosine kinase
JAK: Janus kinase
BID: Bis in die (Latin)
QD: Quaque die (Latin)
TLR: Toll-like receptors
RA: Rheumatoid arthritis
