# Supplementary material for: Target modulation and pharmacokinetics/pharmacodynamics translation of the BTK inhibitor poseltinib for model-informed phase II dose selection
Source: Sci Rep. 2021 Sep 21;11:18671. doi: 10.1038/s41598-021-98255-7 (PMC8455565; doi:10.1038/s41598-021-98255-7)
Supplement: Supplementary file 1 — Supplementary Information. [file 41598_2021_98255_MOESM1_ESM.docx]

**Supplementary Figures**

**Target Modulation, Pharmacokinetics and Pharmacodynamics Translation of the BTK Inhibitor Poseltinib for Model Informed Phase II Dose Selection**

Joo-Yun Byun^1^, Yi T. Koh^2^, Sun Young Jang^1^, Jennifer W. Witcher^2^, Jason R. Chan^2^, Anna Pustilnik^2^, Mark J. Daniels^2^, Young Hoon Kim^1^, Kwee Hyun Suh^1^, Matthew D. Linnik^2^**^#^**, Young-Mi Lee^1^**^#^**

^1^Hanmi Research Center, Hanmi Pharm. Co. Ltd., Hwaseong-si, Republic of Korea

^2^Lilly Biotechnology Center, 10290 Campus Point Drive, San Diego, United States

**Supplementary Table 1**

Kinase selectivity of the Bruton’s tyrosine kinase (Btk) inhibitor, poseltinib and its derivative HM71035 (IC_50_, nM)

|  |  | **Poseltinib** | **HM71035** |
| --- | --- | --- | --- |
| **Biochemical assay** | **BTK** | 1.95 | 3 |
|  | **BMX** | 0.64 |  |
|  | **EGFR** | 4.96 |  |
|  | **JAK3** | 14.6 | 39 |
|  | **ITK** | 103 |  |
|  | **LCK** | >1000 |  |
|  | **LYN** | >1000 |  |
| **Cellular level** | **Cell growth inhibition in EGFR overexppressed A431** | >500 | >500 |
|  | **IL-2 induced T cell proliferation in CTLL-2** | >500 | >500 |

**Supplementary Table 2**.
Antibody Targets used to Measure in Phosphorylation Events

**
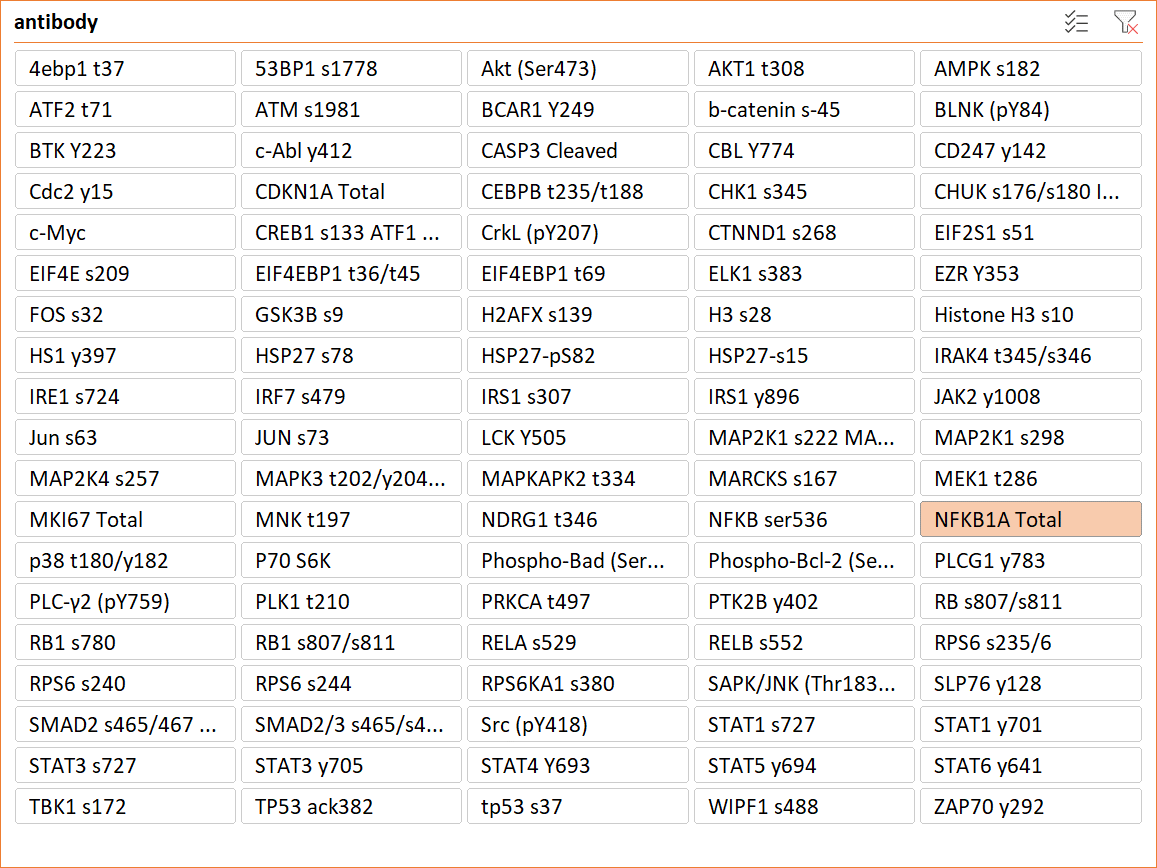
**

**Supplementary Table 3:**

Top Ten Sites with Greatest Difference in Phosphorylation Between Control and Poseltinib (30 µM) and By Cell Type and Stimulation Conditions (Log2Ratio Stim/Control).

Note: Each table includes BTK if BTK was not one of the top 10 sites for difference in phosphorylation

| B cells (IgM+CD40L) 5’ | Site | control | Poseltinib (30 μM) |
| --- | --- | --- | --- |
|  | MAPK3 | 3.73 | 1.30 |
|  | RPS6KA1 | 3.07 | 1.13 |
|  | WIPF1 | 2.75 | 1.81 |
|  | CREB1 | 2.63 | 1.74 |
|  | RPS6 | 2.32 | 1.35 |
|  | Akt | 2.06 | -0.97 |
|  | SAPK/JNK | 1.78 | 0.82 |
|  | PLCγ2 | 1.71 | 0.82 |
|  | SLP76 | 1.58 | 0.86 |
|  | BTK | 1.27 | 0.06 |

| CD8+ Memory (CD3+CD28) 5’ | Site | control | Poseltinib (30 μM) |
| --- | --- | --- | --- |
|  | PLCγ2 | 2.90 | 2.55 |
|  | CrL | 1.97 | 1.59 |
|  | LCK | 1.87 | 1.52 |
|  | BTK | 1.74 | 1.34 |
|  | ZAP70 | 1.71 | 1.33 |
|  | SRC | 1.76 | 0.80 |
|  | WIPF1 | 1.44 | 0.82 |
|  | HS1 | 1.00 | 0.56 |
|  | RPS6KA1 | 0.79 | 0.47 |

| CD4+ Memory (CD3+CD28) 5’ | Site | control | Poseltinib (30 μM) |
| --- | --- | --- | --- |
|  | CD247 | 3.67 | 2.86 |
|  | PLCγ2 | 2.80 | 2.43 |
|  | SLP76 | 1.90 | 1.63 |
|  | CrkL | 1.87 | 1.63 |
|  | LCK | 1.84 | 1.51 |
|  | BTK | 1.55 | 1.21 |
|  | ZAP70 | 1.52 | 1.22 |
|  | PTK2B | 1.37 | 0.93 |
|  | Src | 1.28 | 0.82 |

| NK cells (LPS) 60’ | Site | control | Poseltinib (30 μM) |
| --- | --- | --- | --- |
|  | GSK3B | 1.17 | 0.91 |
|  | SMAD2 | 0.90 | 0.67 |
|  | MAP2K1 | 0.63 | 0.51 |
|  | Akt | 0.54 | 0.54 |
|  | AKT1 | 0.49 | 0.44 |
|  | P38 | 0.42 | 0.36 |
|  | RPS6 | 0.36 | 0.30 |
|  | SMAD2 | 0.27 | 0.07 |
|  | MNK | 0.79 | 0.07 |
|  | BTK | 0.04 | -0.04 |

| Neutrophils (LPS) 15’ | Site | control | Poseltinib (30 μM) |
| --- | --- | --- | --- |
|  | MAP2K1 | 2.18 | 2.38 |
|  | GSK3B | 1.08 | .090 |
|  | RPS6 | 0.91 | 0.66 |
|  | AKT1 | 0.86 | 0.66 |
|  | Akt | 0.85 | 0.66 |
|  | MNK | 0.12 | 0.40 |
|  | 4ebp1 | 0.11 | 0.28 |
|  | AMPK | 0.11 | 0.29 |
|  | HSP27 | 0.07 | 0.34 |
|  | BTK | -0.26 | -0.25 |

| Monocytes (M-CSF)5’ | Site | control | Poseltinib (30 μM) |
| --- | --- | --- | --- |
|  | MAPK3 | 3.21 | 1.88 |
|  | RPS6KA1 | 1.93 | 1.33 |
|  | Akt | 1.78 | 1.06 |
|  | NDRG1 | 1.54 | 0.97 |
|  | HSP27 | 1.28 | 1.80 |
|  | RPS6 | 1.18 | 0.89 |
|  | GSK3B | 1.07 | 0.82 |
|  | RELB | 1.03 | 0.82 |
|  | AKT1 | 0.92 | 0.41 |
|  | BTK | 0.41 | -0.12 |

**Supplementary Table 4**.

Relationship between dose, average predicted occupancy, and predicted average inhibition of relevant pathways.

| **Poseltinib dose** | **Model-predicted receptor occupancy** | **BCR inhibition** | **FcR inhibition** | **TLR inhibition** | **CD3 inhibition** |
| --- | --- | --- | --- | --- | --- |
| 1 mg | 53.2% | 0% | 21.5% | 0% | 11.3% |
| 2 mg | 69.5% | 8.6% | 30.9% | 0% | 17.2% |
| 3 mg | 77.3% | 23.2% | 37.4% | 0% | 21.6% |
| 4 mg | 81.9% | 35.3% | 42.2% | 0% | 25.1% |
| 5 mg | 85.0% | 43.4% | 46.1% | 0% | 28.1% |
| 10 mg | 91.7% | 56.2% | 58.0% | 0% | 38.3% |
| 30 mg | 96.4% | 59.4% | 73.0% | 0% | 55.3% |
| 40 mg | 97.0% | 59.5% | 75.7% | 0% | 59.1% |

Relationship between the dose, predicted occupancy, and predicted inhibitory effect on relevant pathways, such as the B-cell receptor (BCR), Fc receptor (FcR), toll-like receptor (TLR), or CD3 after administration of 1 to 40 mg poseltinib.

**Supplemental Table 5.**

Clinical trials for model calibration and qualification
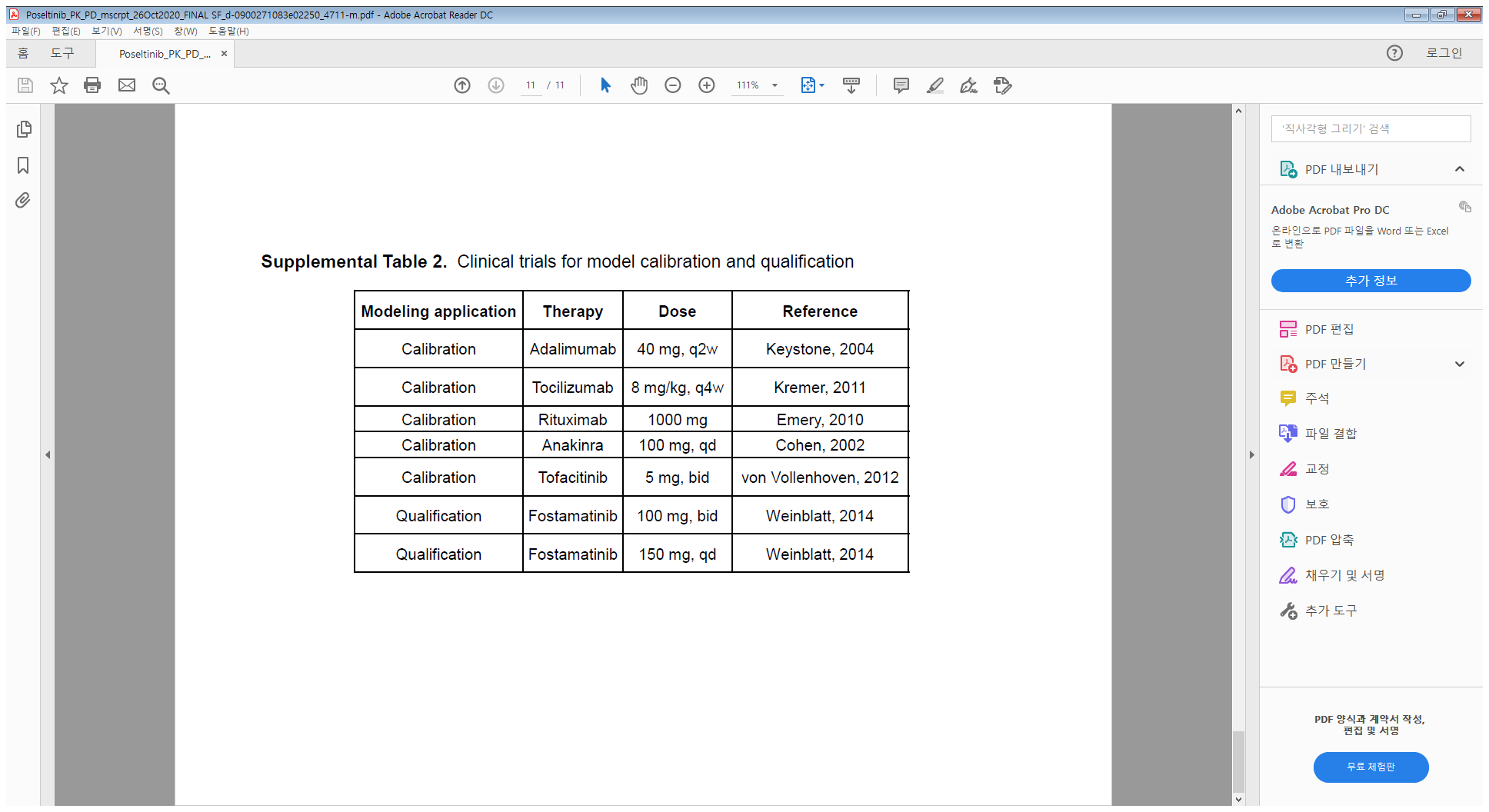


Modeling application models and doses usage for rheumatoid arthritis drugs which are currently in clinical trials for dose selection.

**Supplementary Figure 1**

Full length immunoblot gel for phospho-BTK, total BTK and house keeping protein in PBMCs at day 8 after once-daily oral administration of HM71224 in rats (n=4).

1. Phospho-BTK (Y233)


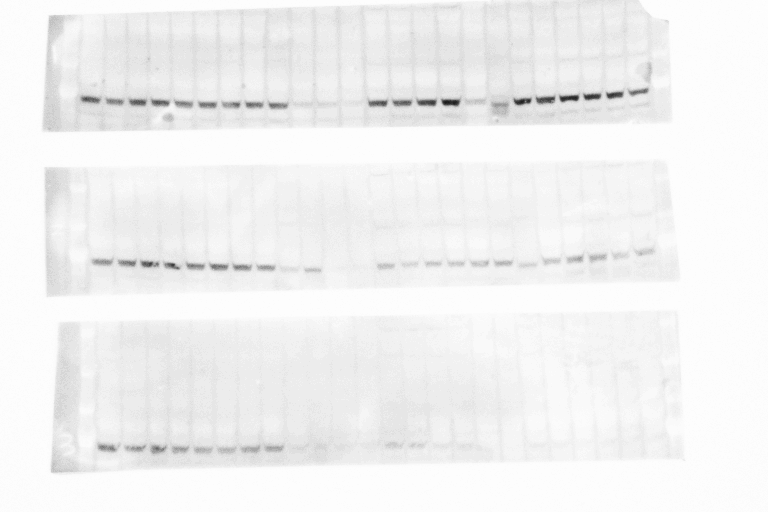


0.3 mg/kg

1 mg/kg

3 mg/kg

75

100

150

250

75

100

150

75

100

150

kDa

Control

0 hr

4 hr

12 hr

24 hr

Control

0 hr

4 hr

12 hr

24 hr

Control

0 hr

4 hr

12 hr

24 hr

1

2

3

4

5

6

7

8

9

10

11

12

13

14

15

16

17

18

19

20

21

22

23

24

1

2

3

4

5

6

7

8

25

26

27

28

29

30

31

32

33

34

35

36

37

38

39

40

1

2

3

4

5

6

7

8

41

42

43

44

45

46

47

48

49

50

51

52

53

54

55

56

1. Total BTK

100


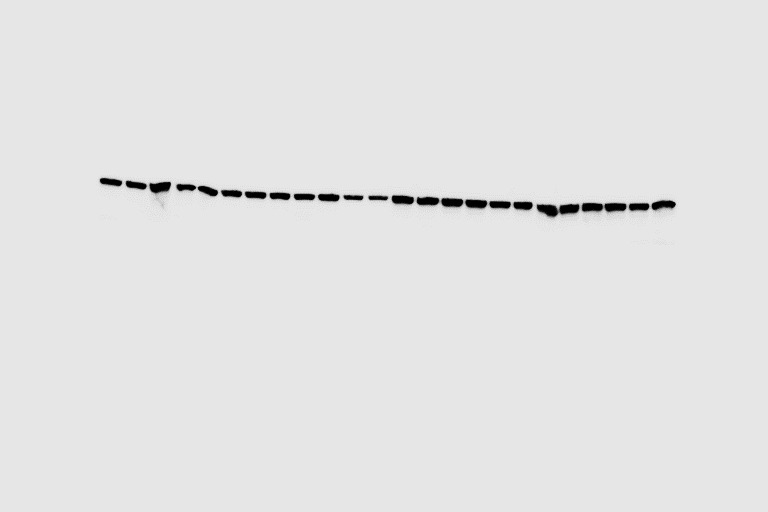


0.3 mg/kg

1 mg/kg

3 mg/kg


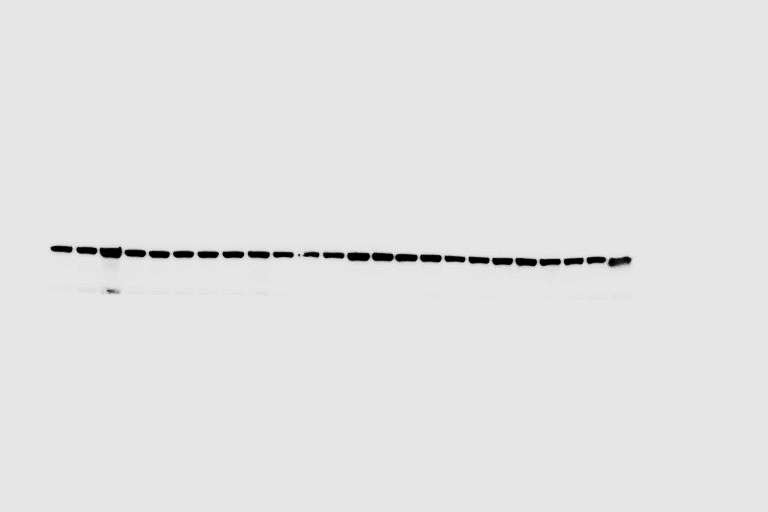

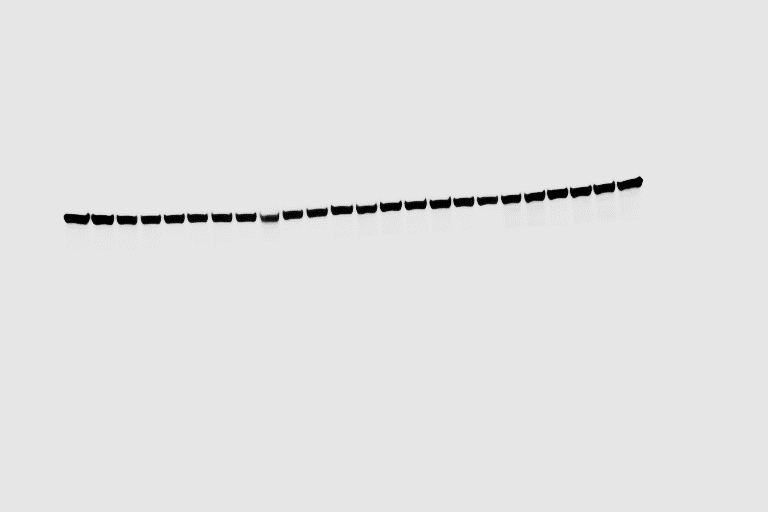


75

50

75

100

50

75

100

50

kDa

kDa

kDa

Control

0 hr

4 hr

12 hr

24 hr

Control

0 hr

4 hr

12 hr

24 hr

Control

0 hr

4 hr

12 hr

24 hr

1

2

3

4

5

6

7

8

9

10

11

12

13

14

15

16

17

18

19

20

21

22

23

24

1

2

3

4

5

6

7

8

25

26

27

28

29

30

31

32

33

34

35

36

37

38

39

40

1

2

3

4

5

6

7

8

41

42

43

44

45

46

47

48

49

50

51

52

53

54

55

56

1. GAPDH


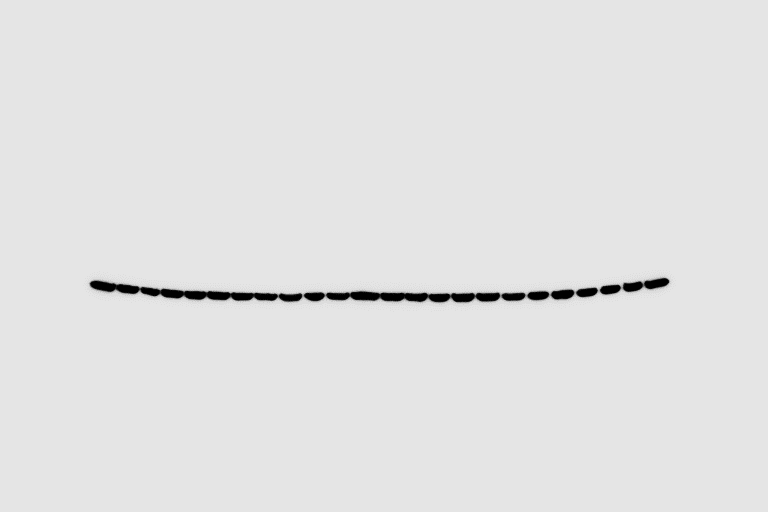

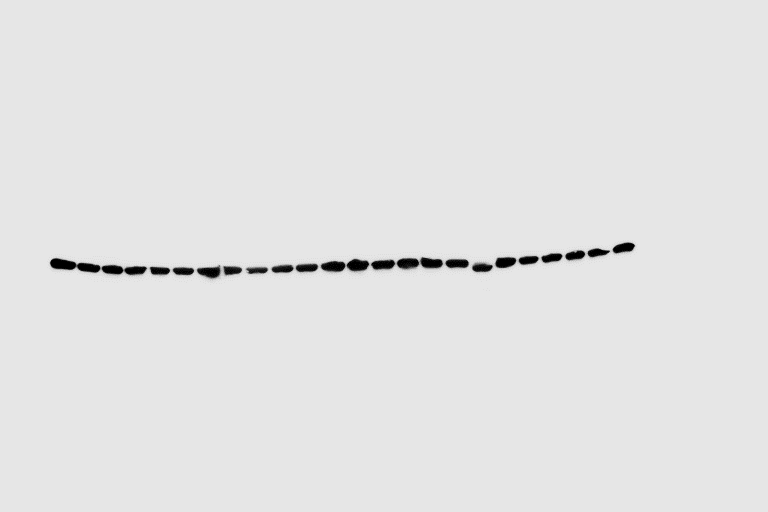

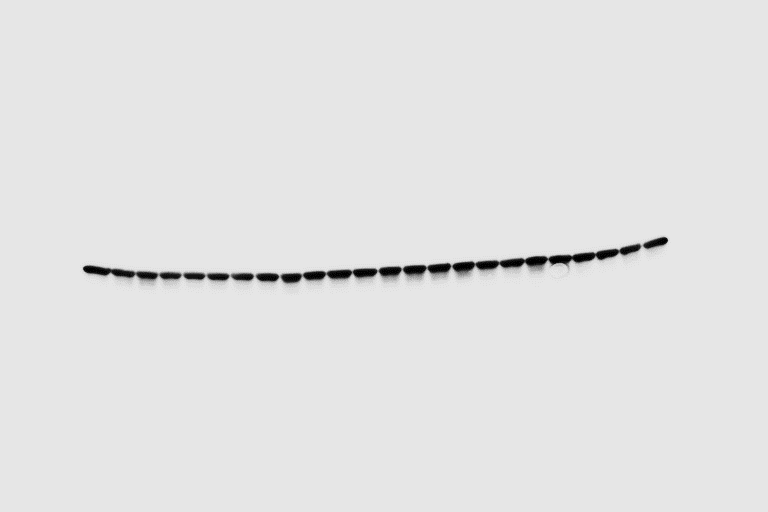


0.3 mg/kg

1 mg/kg

3 mg/kg

37

50

25

kDa

37

50

25

kDa

37

50

25

kDa

Control

0 hr

4 hr

12 hr

24 hr

Control

0 hr

4 hr

12 hr

24 hr

Control

0 hr

4 hr

12 hr

24 hr

1

2

3

4

5

6

7

8

9

10

11

12

13

14

15

16

17

18

19

20

21

22

23

24

1

2

3

4

5

6

7

8

25

26

27

28

29

30

31

32

33

34

35

36

37

38

39

40

1

2

3

4

5

6

7

8

41

42

43

44

45

46

47

48

49

50

51

52

53

54

55

56
